# Cardiac Arrhythmias and Sleep-Disordered Breathing in Patients with Heart Failure

**DOI:** 10.3390/ijms151018693

**Published:** 2014-10-16

**Authors:** Wolfram Grimm, Ulrich Koehler

**Affiliations:** 1 Department of Cardiology, University Hospital of Marburg and Gießen, Philipps-University Marburg, Marburg 35033, Germany; E-Mail: grimmw@med.uni-marburg.de; 2 Sleep Disorder Unit of the Department of Pneumology, University Hospital of Marburg and Gießen, Philipps-University Marburg, Marburg 35033, Germany

**Keywords:** central sleep apnea, cardiac arrhythmias, heart failure, sudden death

## Abstract

The relationship between heart failure (HF), sleep-disordered breathing and cardiac arrhythmias is complex and poorly understood. Whereas the frequency of predominantly obstructive sleep apnea in HF patients is low and similar or moderately higher to that observed in the general population, central sleep apnea (CSA) has been observed in approximately 50% of HF patients, depending on the methods used to detect CSA and patient selection. Despite this high prevalence, it is still unclear whether CSA is merely a marker or an independent risk factor for an adverse prognosis in HF patients and whether CSA is associated with an increased risk for supraventricular as well as ventricular arrhythmias in HF patients. The current review focuses on the relationship between CSA and atrial fibrillation as the most common atrial arrhythmia in HF patients, and on the relationship between CSA and ventricular tachycardia and ventricular fibrillation as the most frequent cause of sudden cardiac death in HF patients.

## 1. Introduction

**Figure 1:**
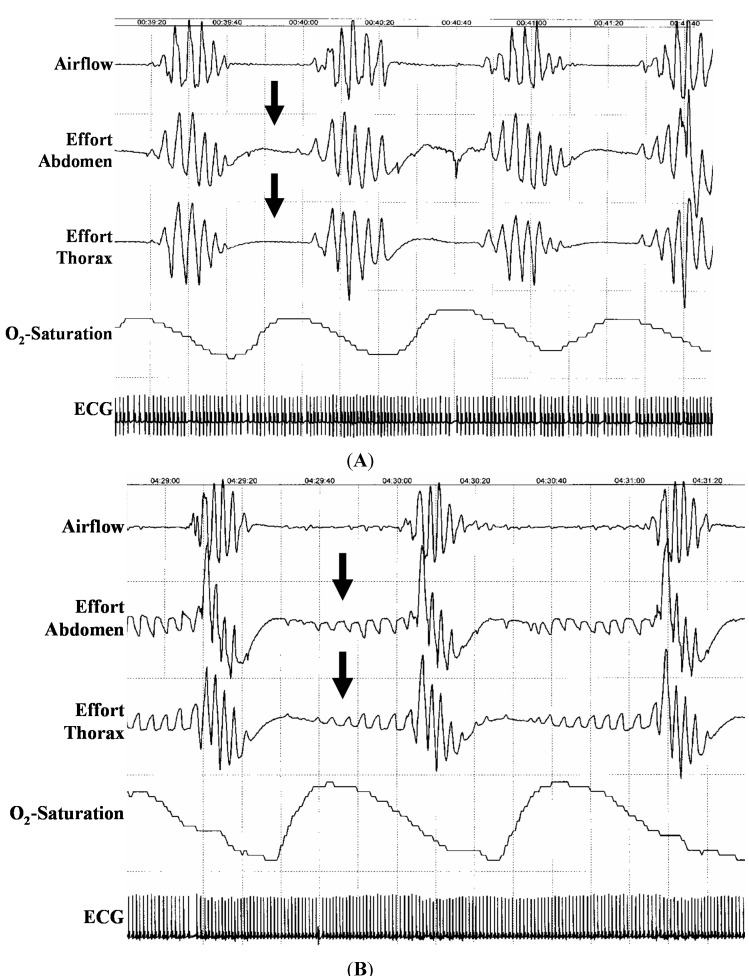
Polysomnography (3 min) with central sleep apnea (**A**) and obstructive sleep apnea (**B**) Note the absence of chest efforts and abdominal movements in the absence of oronasal airflow in central sleep apnea but not in obstructive sleep apnea (arrows). Also, note the pronounced decrease in O_2_-saturation following each apnea episode.

Sleep-disordered breathing and cardiac arrhythmias are both highly prevalent findings in patients with heart failure (HF) [1,2,3,4,5,6,7,8,9,10,11,12,13,14,15,16,17,18,19,20,21,22,23,24,25,26,27,28,29,30]. In contrast to cardiac arrhythmias, which can readily be diagnosed by 12-lead electrocardiogram (ECG) or long-term Holter ECG recordings, screening for sleep-disordered breathing is uncommon in HF patients in the absence of symptoms like excessive daytime sleepiness. Therefore, sleep apnea remains a highly underdiagnosed condition in patients with HF. The frequency of obstructive sleep apnea (OSA) in HF patients is similar or moderately higher to that observed in the general population ranging from 8% in the study by Grimm *et al*. [16], to 38% in the study by Sin *et al.* [1]. This wide range of OSA frequency in HF patients may be explained by differences in methods and patient population. Grimm *et al*. [16] found a low OSA prevalence of 8% by screening HF patients, after patients with a history of sleep disordered breathing and patients who were referred to the sleep laboratory for symptoms suggestive of sleep disordered breathing had been excluded, whereas Sin *et al.* [1], found a high OSA prevalence of 38% in a retrospective study including 450 HF patients, all of whom were referred to the sleep laboratory because of suspected sleep disordered breathing. The majority of previous studies, however, found a much higher prevalence of central sleep apnea (CSA) compared to OSA in patients with HF ranging from 21% to 82% [1,2,3,4,5,6,7,8,9,10,11,12,13,14,15,16,17,18,19,20,21,22,23,24,25,26,27,28,29,30] (Figure 1). This wide range of CSA frequency in HF patients may be explained by a number of variables including HF severity and etiology, age, gender, and HF medication. In addition, apnea-hypopnea index cut-off values used to define CSA vary considerably between 5/h [3,18], 15/h [1,7,17] and 30/h [16]. For the purpose of this review, we use an apnea-hypopnea index (AHI) <15/h to define no or mild sleep apnea, an AHI ≥15/h but ≤30/h to define moderate apnea, and an AHI cutoff point >30/h to define severe sleep apnea as recommended by the task force of the American Society of Sleep Medicine unless specified otherwise [25]. In addition, we focus in this review on HF with reduced ejection fraction rather than HF with preserved ejection fraction, *i.e.*, diastolic heart failure, because there is a large body of evidence in the literature with regard to the relationship between arrhythmias, sleep disordered breathing and heart failure with reduced ejection fraction, whereas the relationship between arrhythmias, sleep disordered breathing and diastolic dysfunction with in HF patients with preserved ejection fraction is largely unknown [12,21,23,27].

**Figure 2:**
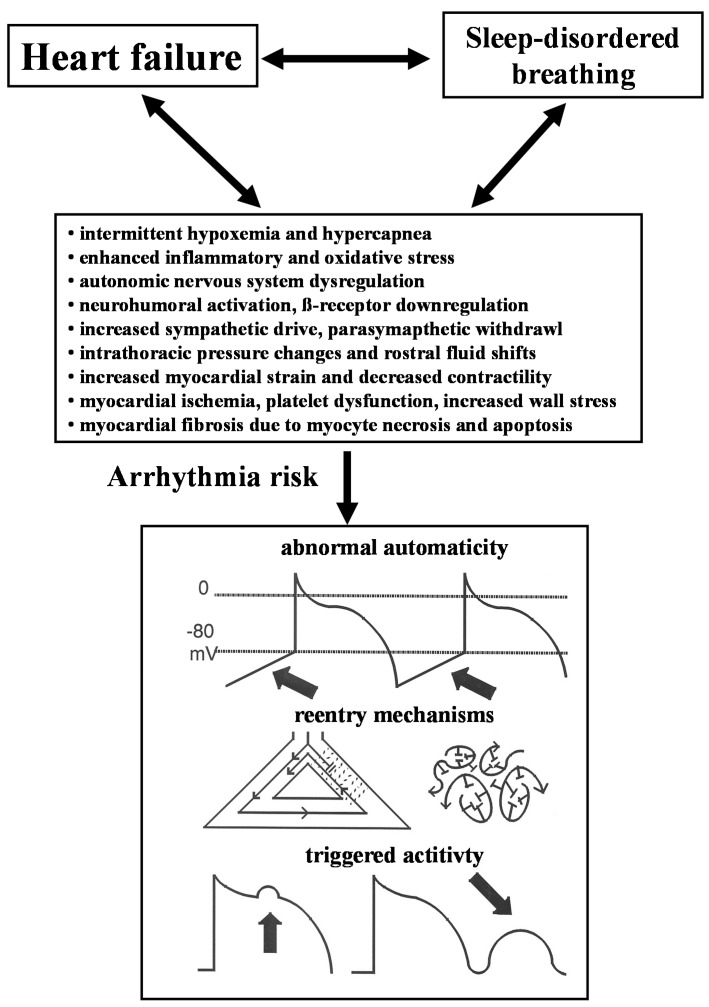
Relationship between heart failure, sleep-disordered breathing and cardiac arrhythmias.

**Figure 3:**
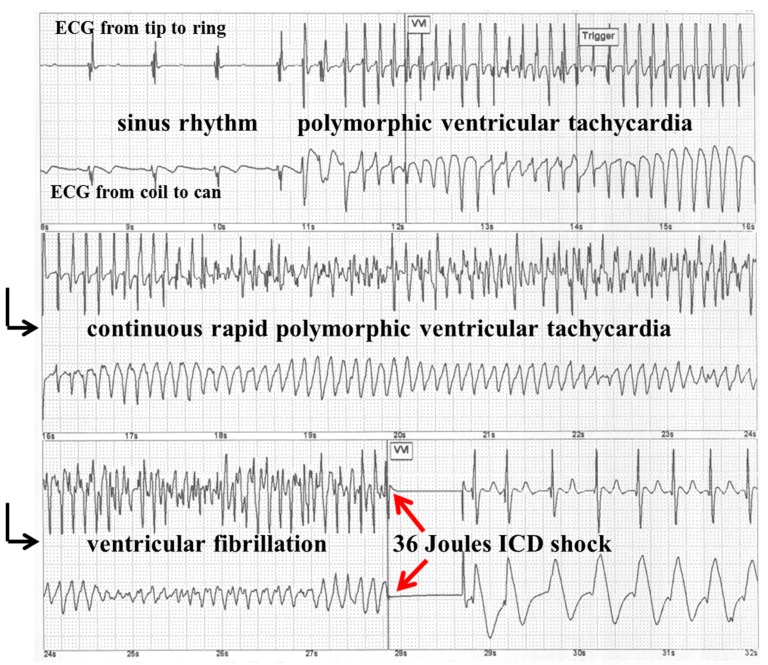
Stored implantable cardioverter defibrillator (ICD) electrocardiogram (ECG) showing a spontaneous episode of rapid polymorphic ventricular tachycardia, which is terminated after 9 s by the first 36 Joules ICD shock. The black arrows indicate that it is a continuous ECG tracing. The red arrows mark the termination of ventricular fibrillation by a 36 Joules ICD shock.

The purpose of this review is threefold. First, we will briefly summarize our knowledge about the complex interaction between heart failure, sleep-disordered breathing and cardiac arrhythmias (Figure 2). Second, we describe the association between sleep-disordered breathing and atrial fibrillation as the most common atrial arrhythmia in HF. Finally, we summarize our current knowledge regarding the clinically important question whether sleep-disordered breathing is merely a marker or an independent risk factor for sustained ventricular tachycardia or ventricular fibrillation as the most frequent cause of sudden cardiac deaths in HF patients [31,32,33,34,35,36,37,38,39,40,41,42] (Figure 3).

## 2. Interaction between Heart Failure, Sleep-Disordered Breathing and Cardiac Arrhythmias

Both prevalence and CSA severity have been associated with increased arrhythmogenic risk synergistic to HF severity with increased neurohumoral activation, higher brain natriuretic peptide (BNP) levels, increased pulmonary capillary wedge pressure, and lower ejection fraction [21,23,25,26] (Figure 2). Heart failure leads to increased left ventricular (LV) wall stress and increased LV filling pressures resulting in pulmonary congestion, which subsequently activates lung irritant vagal receptors. The Cheyne-Stokes respiration pattern in HF patients of alternating hyperventilation and apnea is sustained by a complex interaction of pulmonary congestion due central fluid accumulation triggered by increased venous return in the supine position, increased respiratory chemoreceptor drive, apnea-induced hypoxemia, and arousals, which trigger oscillations in the arterial carbon dioxide level above and below the central threshold of ventilation, termed the apneic threshold. Intermittent arousals cause further abrupt increases in ventilation und decrease the arterial carbon dioxide level below the threshold for ventilation, triggering the next central apnea episode. Although CSA episodes have a different pathophysiology than OSA without generating an exaggerated negative intrathoracic pressure, both CSA and OSA increase sympathetic nervous system activity in HF patients accompanied by various neurohumoral and hemodynamic responses that together further stress the failing heart. The apneas provoke periodic elevations in sympathetic activity and parasympathetic withdrawal [26], favoring atrial and ventricular irritability due to abnormal automaticity and triggered activity (Figure 2). Increased sympathetic activity may subsequently contribute to tachycardia, peripheral vasoconstriction, and activation of the renin-angiotensin system with increased oxygen demand, blood pressure, blood volume, and myocardial oxygen demand. This chain of events may finally contribute to a pathophysiological vicious circle [27]. Increased inspiratory efforts between apnea episodes are accompanied by lower intrathoracic pressures which also contribute to increased left ventricular pressure, increased wall stress and afterload. In addition, sleep disordered breathing in HF patients has been linked to increased levels of various markers of inflammation, oxidative stress, endothelial dysfunction, platelet dysfunction, and myocardial ischemia, which may cause myocyte necrosis and apoptosis, and subsequently increase the amount of myocardial myocardial fibrosis in the atria as well as in the ventricles. Diffuse myocardial fibrosis in both atria facilitate reentry by multiple wavelets as the most common mechanism of persistent atrial fibrillation, whereas atrial fibrillation is most often triggered by supraventricular premature beats originating in one or more pulmonary veins. This observation led to the concept of pulmonary vein isolation using radiofrequency energy or cryoablation in patients with paroxysmal atrial fibrillation. On the ventricular level, more or less diffuse myocardial fibrosis has also been recognized to be the substrate for reentrant ventricular tachyarrhythmias, the most frequent mechanism of sudden death in HF patients (Figure 2).

## 3. Sleep-Disordered Breathing and Atrial Fibrillation in Heart Failure

Obstructive sleep apnea has consistently been shown to be an independent risk factor for atrial fibrillation in patients with and without heart failure [19,20,21]. In addition, Kanagala and coworkers [22] observed a higher recurrence rate of atrial fibrillation after cardioversion in patients with untreated OSA compared to patients with continuous positive airway pressure (CPAP) treated OSA. In contrast to OSA, the relationship between CSA and atrial fibrillation remains controversial. The results of 14 studies investigating the association between CSA and atrial fibrillation in at least 100 HF patients are summarized in Table 1. These studies reported a high prevalence of CSA in patients with left ventricular systolic dysfunction, ranging from 25% to 64%. This wide range of CSA frequency in heart failure patients may be explained by a number of variables including patient selection, heart failure severity and etiology, age, gender, heart failure medication and various AHI cut-off values used to define CSA as mentioned above. Unfortunately, the results of previous studies investigating the association between atrial fibrillation (AF) and CSA in patients with LV systolic dysfunction are contradictory (Table 1). Six studies found a similar prevalence of atrial fibrillation in patients with *versus* without CSA [2,9,11,15,16,18]. Another 8 studies, however found a significantly higher prevalence of atrial fibrillation in patients with *versus* without CSA [1,3,6,7,12,13,19,24]. A prospective observational study at our institution [17] enrolled 267 patients with chronic stable HF, who were screened for sleep disordered breathing using cardiorespiratory polysomnography, after patients with predominantly obstructive sleep apnea or insufficient sleep studies had been excluded. We found atrial fibrillation at study entry in 26% of 267 patients. CSA with an AHI ≥15/h was present in 43% of 267 patients and 25% of 267 patients had severe CSA with an AHI >30/h. Multivariate analysis revealed a significant association between atrial fibrillation and severe CSA (odds ratio (OR): 5.21; 95% CI: 1.67–16.27, *p* = 0.01), age (OR: 1.22 per 5-year increase; 95% CI: 1.05–1.40, *p* = 0.01), left atrial diameter (OR 1.61 per 5 mm increase; 95% CI: 1.22–2.01, *p* < 0.01) and digitalis (OR: 2.7; 95% CI: 1.26–5.79, *p* = 0.01). Thus, the results our study [17] suggest that atrial fibrillation is associated with severe CSA, but not with moderate CSA in addition to age, use of digitalis and left atrial size in patients with LV systolic dysfunction. Similar to our study [17], only one previous study [1] reported the relation between atrial fibrillation and CSA in HF patients using multivariate analysis. Sin and colleagues [1] found atrial fibrillation, male gender, and age >60 years to be independent risk factors for CSA using an AHI cutoff >10/h to diagnose sleep apnea (Table 1). In contrast to our study [17], patients were enrolled more than 15 years ago in the study by Sin *et al.* [1], and, therefore, did not routinely receive modern heart failure therapy including β-blockers or aldosterone antagonists in addition to angiotensin converting enzyme (ACE) inhibitors or angiotensin receptor blockers (ARBs). Furthermore, 13% of heart failure patients in our study received cardiac resynchronization therapy, which was also not available in the study of Sin *et al.* [1]. A metaanalysis by Lamba *et al.* [23], found a substantial improvement of systolic LV function by cardiac resynchronization therapy, which was accompanied by a significant decrease in sleep apnea severity in CSA patients with a mean AHI reduction of 13/h. In contrast to cardiac resynchronization therapy in HF patients with CSA, cardiac resynchronization therapy did not result in a significant AHI decrease in HF patients with OSA, which may be explained by the different pathophysiology of OSA *versus* CSA.

Mehra and coworkers [3] analyzed nocturnal arrhythmias in 2 samples of the Sleep Heart Health Study consisting of a sample of 228 subjects with severe sleep-disordered breathing with an AHI ≥30/h compared to an age-, sex-, ethnicity-matched sample of 338 subjects without sleep apnea with an AHI <5/h. Similar to our study [17], Mehra *et al.* [3], found a significantly higher prevalence of atrial fibrillation in patients with *versus* without sleep-disordered breathing (4.8% *versus* 0.9%). In contrast to our study, however, only 3% of patients in the study by Mehra and colleagues [3] had heart failure due to the design of the Sleep Heart Health Study as a community-based epidemiologic study in participants aged ≥40 years. In summary, our prospective study [17] strongly suggests a significant association between AF and severe CSA but not with mild or moderate CSA in HF patients. In contrast to patients with severe obstructive sleep apnea, CPAP therapy failed to improve the outcome of HF patients with CSA in the prospective randomized CANPAP trial [28]. More recently, several small studies have shown improvements of left ventricular function with adaptive servo-ventilation for severe CSA, which has the greater ability to normalize the breathing pattern in HF patients with CSA compared to CPAP ventilation [29,30]. Future studies need to clarify whether adaptive servo-ventilation for severe CSA is helpful to prevent atrial fibrillation and to improve symptoms and decrease mortality in HF patients.

## 4. Sleep-Disordered Breathing and Sudden Cardiac Death in Heart Failure

CSA including Cheyne-Stokes’ respiration in HF patients has been be associated with increased risk for life-threatening ventricular arrhythmias and sudden death, although most studies investigating the relation between cardiac arrhythmias and sleep-disordered breathing in HF were small and yielded conflicting results [31,32,33,34,35,36,37]. In the last decade, implantable cardioverter-defibrillators (ICDs) have become the therapy of first choice to prevent sudden death in high risk patients for life-threatening ventricular tachycardia or ventricular fibrillation. Most patients with ICD suffer from congestive heart failure due to ischemic or nonischemic cardiomyopathy. Since all modern ICD systems have automatic ECG storage capability for spontaneous arrhythmias triggering ICD therapy, the association between sleep-disordered breathing and life threatening sustained ventricular tachycardia or ventricular fibrillation as documented by stored ICD-ECGs can easily be determined in ICD patient populations [2,16,18,38,39,40,41,42] (Table 2, Figure 3).

We recently performed a large prospective observational study [31] to assess the prognostic significance of sleep-disordered breathing in ICD recipients with regard to total mortality and appropriate ICD therapy for ventricular tachycardia or fibrillation. In this study [31], we enrolled 204 ICD patients without known sleep apnea, 51% of whom had CSA and 14% had OSA (Table 2). As a result, age, LV end-diastolic diameter, secondary prevention ICD indication, use of diuretics and absence of aldosterone antagonists were independently associated with appropriate ICD therapy, whereas decreased left ventricular ejection fraction and increased age were independent predictors of total mortality [31]. The presence and severity of previously unknown sleep apnea were not independently associated with appropriate ICD therapy or with total mortality [31]. Similar to the results of our study [31], Fries *et al.* [38], and Staniforth *et al.* [2], found no association between sleep-disordered breathing and appropriate ICD therapy for VT or VF in two smaller cohorts including of 40 patients and 101 patients, respectively. Three previous studies [40,41,42], however, found a higher incidence of appropriate ICD therapy in patients with *versus* without sleep disordered breathing. All three studies, however, were limited by small cohorts of 22 patients [41], 45 patients [42] and 71 patients [40], respectively. Recently, Bitter *et al.* [16], reported a series of 255 patients who were screened for sleep disordered breathing 6 months after implantation of a cardiac resynchronization device combined with ICD. By multivariate Cox analysis, Bitter *et al.* [16], found an independent association between OSA and CSA and appropriate ICD therapies during 48 months of follow-up (Table 2). The discrepancy between the study by Bitter *et al.* [16], and our study [31] may be in part because of differences in methods and patient selection. In contrast to our study, Bitter *et al.* [16], performed sleep studies in 472 patients 6 months after implantation of a cardiac resynchronization device, with subsequent exclusion of 182 patients with moderate to severe sleep apnea who received recommended ventilation therapy during follow-up. Thus, Bitter *et al.* [16], reported exclusively the results of patients with moderate to severe sleep disordered breathing who refused to receive ventilation therapy and patients with mild or no sleep apnea. In contrast to the study of Bitter *et al.* [16], ventilation therapy for moderate-to-severe sleep apnea was not part of our study protocol [31] and was not performed in 98% of patients during follow-up, because, in the CANPAP trial, [28] ventilation therapy using CPAP has not been shown to improve prognosis.

**Table 1:** Association between atrial fibrillation and CSA in studies with at least 100 patients with heart failure.

First Author	Year	Patients, *n*	LVEF, %	CSA, %	CSA & AF, *%*	SA & AF, *%*	*p* Univariate	*p* Multivariate	OR (95% CI)
Sin [1]	1999	450	23 ± 16	33	23	8	<0.05	<0.05	4.13 (1.53–11.14)
Staniforth [2]	2005	101	33	41	19	8	n.s.	–	–
Mehra [3]	2006	566	n.a.	40 *	5	1	0.003	<0.05	4.02 (1.03–15.74)
Oldenburg [6]	2007	700	28 ± 7	40	35	14	<0.05	–	–
Schulz [7]	2007	203	28 ± 1	28	44	22	<0.05	–	–
Christ [9]	2007	102	28 ± 10	37	26	14	n.s.	–	–
Oldenburg [11]	2008	105	≤40	58	23	30	n.s.	–	–
Bitter [12]	2009	244	>55	30	24	17	<0.05	–	–
Paulnio [13]	2009	316	30 ± 11	25	29	12	<0.05	–	–
Jilek [15]	2011	296	≤50	64 *	13	20	n.s.	–	–
Mehra [24]	2009	2911	n.a. **	n.a.	15	3	<0.05	<0.05	2.69 (1.61–4.47)
Bitter [16]	2011	255	<40	27	12	12	n.s.	–	–
Kreuz [18]	2013	133	≤35	62 *	30	27	n.s.	–	–
Grimm [17]	2014	267	34 ± 10	25	39	22	0.002	0.01	5.21 (1.67–16.27)

* Including patients with obstructive sleep apnea; ** Outcome of Sleep Disorders in Older Men (MrOS Sleep) Study [24] with 6% prevalence for self-reported heart failure; LVEF = left ventricular ejection fraction; CSA = central sleep apnea; AF = atrial fibrillation; SA = sleep apnea; OR = odds ration; CI = confidence interval; n.s. = not significant; n.a. = not available; *n* = number of patients.

**Table 2:** Association CSA and sustained ventricular tachycardia in studies with at least 70 ICD patients.

First Author	Year	Patients, *n*	LVEF, *%*	SDB, *n* (%)	CSA, *n* (%)	OSA, *n* (%)	Follow-Up, Months	SA & VT, *n* (%)	SA & VT, *n* (%)	*p* Univariate	*p* Multivariate
Staniforth [2]	2005	101	33	42 (42)	42 (42)	n.a.	6	11 (26)	15 (25)	1.0	n.s.
Serizawa [4]	2008	71	31	47 (66)	n.a.	n.a.	6 ± 0	20 (43)	4 (17)	0.03	0.02
Bitter [16]	2011	255	n.a.	169 (66)	87 (34)	82 (32)	48	103 (61)	37 (43)	<0.01	<0.05
Grimm [31]	2012	204	29 ± 10	134 (66)	105 (51)	29 (14)	38 ± 26	56 (42)	24 (34)	0.56	n.s.
Kreutz [18]	2013	133	27 ± 5	82 (62)	n.a.	n.a.	24 ± 8	44 (54)	17 (34)	0.03	0.01

CSA = central sleep apnea; LVEF = left ventricular ejection fraction; *n* = number of patients; n.a. = not available; n.s. = not significant; OSA = obstructive sleep apnea; SA = sleep apnea; SDB = sleep disordered breathing; VT = ventricular tachycardia.

## 5. Conclusions

There is consensus in the literature that undiagnosed sleep-disordered breathing, especially CSA, is common in HF patients. A recent study [17] strongly suggests that atrial fibrillation is independently associated with severe CSA in addition to age and left atrial size in HF patients. The prognostic significance of sleep-disordered breathing with regard to sustained ventricular tachycardia or ventricular fibrillation in HF patients with ICD, however, remains controversial, as summarized in Table 2. A prospective observational study including 204 ICD recipients at our institution [31] strongly suggests that the presence and severity of previously unknown sleep apnea in ICD recipients is not an independent predictor of appropriate ICD therapy or mortality during follow-up.
